# A Network Flow-Based Method to Predict Anticancer Drug Sensitivity

**DOI:** 10.1371/journal.pone.0127380

**Published:** 2015-05-18

**Authors:** Yufang Qin, Ming Chen, Haiyun Wang, Xiaoqi Zheng

**Affiliations:** 1 College of Information Technology, Shanghai Ocean University, Shanghai, China; 2 Department of Bioinformatics, School of Life Science and Technology, Tongji University, Shanghai, China; 3 Department of Mathematics, Shanghai Normal University, Shanghai, China; Southern Illinois University School of Medicine, UNITED STATES

## Abstract

Predicting anticancer drug sensitivity can enhance the ability to individualize patient treatment, thus making development of cancer therapies more effective and safe. In this paper, we present a new network flow-based method, which utilizes the topological structure of pathways, for predicting anticancer drug sensitivities. Mutations and copy number alterations of cancer-related genes are assumed to change the pathway activity, and pathway activity difference before and after drug treatment is used as a measure of drug response. In our model, Contributions from different genetic alterations are considered as free parameters, which are optimized by the drug response data from the Cancer Genome Project (CGP). 10-fold cross validation on CGP data set showed that our model achieved comparable prediction results with existing elastic net model using much less input features.

## Introduction

Systematical depiction of the relationships between genetic alterations and cancer vulnerabilities is a major goal for current cancer genome projects. Cancers are induced by the accumulation of genetic alterations within a cell, including inherited genetic mutations, chromosome translocations, and copy number alterations [1]. Association analysis between genetic alterations and anticancer drug sensitivity could provide new insights for biomarker discovery and drug sensitivity predictions. However, the huge diversity of different cancer types, even in tumors from the same tissue, makes the above aim very challenging.

Much effort has been made to elucidate biomarkers for anticancer drugs ever since the outcome of high-throughput genomic technique, and most of which are based on gene expression data. For example, Staunton et al. [2] proposed a weighted voting classification strategy to predict a binary response (sensitive or resistant) based on the NCI-60 gene expression data. Based on the same data set, Riddick et al. built an ensemble regression model using Random Forest [3]. Lee et al. developed a coexpression extrapolation algorithm to infer drug signature by comparing differential gene expression between sensitive and resistant cell lines [4]. Due to the heterogeneity of cancers, a biomarker for a drug will be different for different cancer types, so some researchers tend to a certain specific cancer types [5, 6]. For example, Holleman et al. investigated gene expression patterns in drug-resistant acute lymphoblastic leukemia cells and found that combined drug-resistance gene-expression score is significantly associated with the risk of relapse [7]. Besides gene expression, other researchers focus on the relationships between chemotherapy sensitivity and epigenetic modifications, including phosphorylation and methylation. For example, Shen et al. used CpG island methylation profile to predict drug sensitivities in the NCI-60 cancer cell line panel [8]. They got a list of methylation markers that predict sensitivity to chemotherapeutic drugs, e.g., hyper-methylation of the p53 homologue p73 was strongly correlated with sensitivity to alkylating agents. Despite the success in identifying several drug biomarkers, the previously described methods suffer from a limited number of samples (cell lines) compared with the large number of expression genes and chemical compounds used (>100,000). By chance, the gene signature for some compounds may be over-estimated.

Recently, researchers from the Broad and Sanger Institutes generated a large scale genomic data set for more than 1,000 human tumor cell lines, including mutation status, copy number variance, expression profile, and translocation of a selected set of cancer driver genes, as well as the pharmacological profiles for a large number of anticancer drugs [9, 10]. To elucidate the interactions of genomic instabilities with respect to cancer cell drug sensitivity, they applied a so-called elastic net regression to infer sensitivity for each drug by different types of genomic instability data. Though achieving good performance for certain drugs and cancer types, the above method also suffers from the following limitations. First, compared to the huge number of genomic features, the number of cell lines is still not large enough. This kind of learning problem is prone to be over fitting and thus has bad generalization ability, i.e., expressions of some genes may highly correlate with response of a drug only by chance. Second, genes are not independent with each other in expression, but form a certain hierarchical structure, i.e., pathway or PPI network. Unfortunately all of the above methods do not take this information into consideration. Explicitly, most drugs target specifically to genes from some particular pathways that abrogate a variety of cancer-related stressors including DNA damage replication, proteotoxic stress, mitotic stress, and metabolic stress, etc. [11]. Thus, mutation and expression of these genes and their relationships with other genes, especially cancer driver genes within a pathway, would offer better hints for drug sensitivity prediction.

To overcome the above problems, we propose a network flow-based method to predict anticancer drug sensitivity using topological structure of pathways. In our model, mutations and copy number alternations of cancer related genes are assumed to determine its pathway activity. Treated drugs would reduce a certain amount of pathway activity flowing to its target genes. Drug sensitivity of a sample is measured by pathway activity change before and after drug treatment. Based on these assumptions, we come up with an optimization model to fit all parameters based on training samples. As an example, our model achieved good performance for drugs targeting the MAPK pathway through a 10-fold cross-validation. Our algorithm is also applicable to predict combination effects of two or more drugs.

## Material and Methods

### 2.1 Data sources

The cancer genome and drug sensitivity data are available from the Cancer Genome Project (CGP) [10] (S1 Table). This dataset cataloged mutation statuses of 64 commonly mutated cancer genes, genome-wide DNA copy number variance and expression profiles of 14,500 genes in 639 human tumor cell lines, and pharmacological responses for 130 selected anticancer drugs. This data allows systematical discovery of biomarkers or signatures able to characterize, classify, and prognosticate clinical behavior of human tumors. Here we considered only nonsense mutations in coding exon regions and copy number gain and loss of cancer driver genes. Cell viability was assessed from the changes in total cellular protein after 72 hours of drug treatment. Drug sensitivities are evaluated by the half maximal inhibitory concentration (IC50) relative to the control.

During oncogenesis, gene mutations or expression changes accumulate in some pathways regulating specific aspects of cell proliferation. Cancer-related pathways are those pathways whose dysregulation allows cells to grow and divide unchecked including apoptosis, cell cycle, DNA damage repair, and growth factor responses. Here 12 cancer-related pathways were chosen from KEGG database [12]. It is found that genes in these pathways either have genetic aberration or are synthetic lethal, and they additionally play important roles in cell response to chemotherapy drugs [13]. These pathways consist of a total of 909 genes and 5180 interactions (edges), where 43 of 64 cancer genes in CGP are included. Detailed information of these 12 pathways is shown in Table 1.

**Table 1:** 12 important cancer pathways from KEGG.

KEGG_ID	Pathway name	# Gene	# Edge	# Cancer gene
00970	Aminoacyl-tRNA biosynthesis	7	5	0
04010	MAPK signaling pathway	256	875	13
04064	NF-kappa B signaling pathway	80	168	0
04110	Cell Cycle	124	630	13
04115	p53 signaling pathway	68	84	10
04210	Apoptosis	86	183	4
04350	TGF-beta signaling pathway	83	220	3
04330	NOTCH_SIGNALING_PATHWAY	47	138	2
04310	WNT_SIGNALING_PATHWAY	144	775	9
05200	Cancer pathway	307	1104	32
04630	JAK_STAT_SIGNALING_PATHWAY	99	868	10
04340	HEDGEHOG_SIGNALING_PATHWAY	51	130	3

### 2.2 Pathway activity and drug sensitivity prediction

Our algorithm is based on the following three main assumptions: i) Mutations and copy number alternations of cancer genes could affect (increase or decrease) their corresponding pathway activity, with the contributions of different genes performing independent of each other; ii) Cancer drugs with specific target genes reduce a certain percentage of activity passing to their target genes; iii) Pathway activity is defined as the sum of activities of all 12 cancer-related pathways, and its difference before and after drug treatment is used as a measure of drug response.

**Fig 1:**
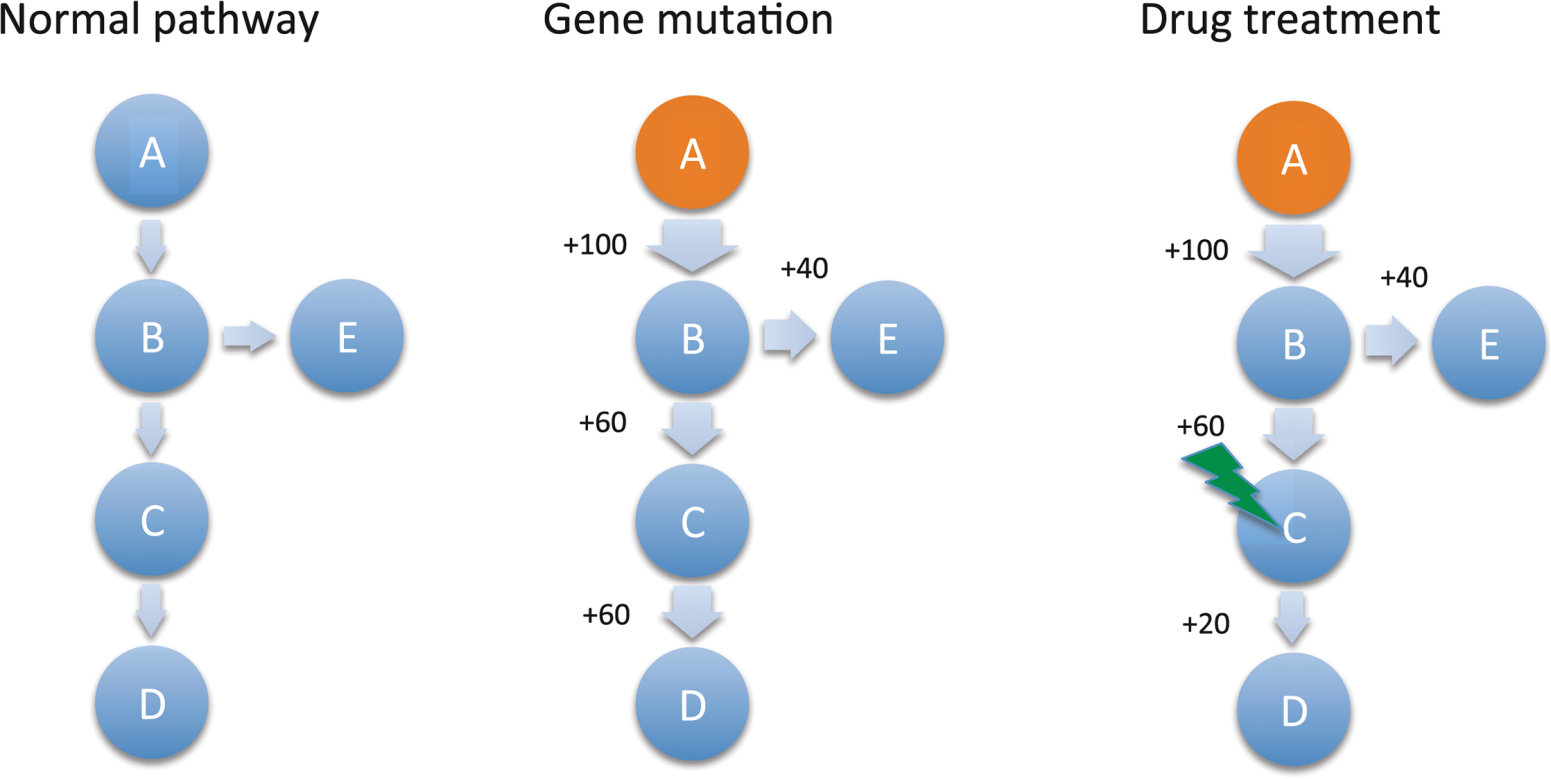
The overall workflow of our algorithm. Left panel lists the mutation status and activity flow of a normal pathway, where width of an edge represents activity flowing from the starting to the ending nodes. If gene A in the pathway mutated (marked as red), we suppose that this will increase the downstream pathway activity by 100, then the increased activity will flow to B, C, D and the terminal nodes eventually. If there are multiple paths from B, each path will share a certain proportion of the increased activity flowing to B. If an inhibitor for gene C is introduced to treat this sample, a certain proportion of increased pathway activity flowing through C will be inhibited. For example, only +20 activity is left after drug treatment.

The overall workflow of our algorithm is summarized in Fig 1. The left panel lists the mutation status and activity flow of a normal pathway, where width of an edge represents activity flowing from the start to the end nodes. If gene A in the pathway is mutant (marked as red, Fig 1B), which is assumed to increase the downstream pathway activity by 100. Then, the increased activity will flow to B, C, D, and eventually the terminal nodes. If there are multiple paths from B, each path will share a certain proportion of the increased activity flowing to B. If a gene C inhibitor is used to treat this sample, the increased pathway activity flowing through C will be partially inhibited. An example is shown in Fig 1C, i.e., only +20 activity is left after drug treatment. So the final output activities before and after drug treatment will have difference, which measures effect of the treated drug. However, in real cases, mutation and copy number statuses of a sample is rather complicated, e.g., there may be more than one mutant gene in a sample, and activity contribution of different cancer genes may be different. Thus, a systematic and quantitative model is needed for better interpreting the real biological phenomenon.

We denote *P*_*i*_ to represent a pathway, and *A*_*i*_ to be activity of this pathway *i* = 1,2,…,12. For each gene *g*_*ij*_ in *P*_*i*_, its mutation and copy number statuses are denoted by and *v*_*ij*_, respectively. Suppose that if this gene is mutant or has abnormal copy number, the pathway activity will increase by $A_{{}_{ij}}^{m}$ and $A_{{}_{ij}}^{v}$, respectively. Mutation of a gene is prone to increase its pathway activity, but only part of the increased activity could “flow” to the target genes of a drug. We termed the fraction of increased activity that can be affected by this drug as “drug influence coefficient”, and denoted as *ρ*_*ij*_. In the ideal case, *ρ*_*ij*_ will close to 1 if drug target gene is in the downstream of *g*_*ij*_ and lies on the only way from *g*_*ij*_ to the terminals, while less than 1 if there are multiple downstream paths from *g*_*ij*_, and only part of (or none of) them pass through the drug target. A drug *d* will inhibit the activity passing through its target genes to a certain percent off, which is referred to be “drug effect” and denoted by *α*.

So for a given sample *s*, the increased pathway activity due to the genetic alteration before drug treatment is

$$AI=\sum_{i=1}^{12}A_{i}=\sum_{i=1}^{12}\sum_{j}m_{ij}A_{{}_{ij}}^{m}+v_{ij}A_{{}_{ij}}^{v}.$$ (1)

After drug treatment, the increased pathway activity is formulated as

$$AT=\sum_{i=1}^{12}A_{}^{}=\sum_{i=1}^{12}\sum_{j}\left(m_{ij}A_{{}_{ij}}^{m}+v_{ij}A_{{}_{ij}}^{v}\right)\rho_{ij}\alpha .$$ (2)

Under the assumption that pathway activity difference before and after drug treatment measures the response of a drug, as the simplest case, we assume the relationship is linear, that is

ΔA=AI−AT=k⋅sens+b, (3)

So

$$\sum_{i=1}^{12}\sum_{j}\left(m_{ij}A_{{}_{ij}}^{m}+v_{ij}A_{{}_{ij}}^{v}\right)\left(1-\rho_{ij}\alpha \right)=k\cdot sens+b.$$ (4)

Denote pathway activity difference of the sample *s* treated with drug *d* as Δ*A* ^(*s*,*d*)^ and the corresponding drug sensitivity as *sens* ^(*s*,*d*)^. The optimal parameters could be obtained by minimizing the sum of prediction errors for all sample-drug combinations,

$$\hat{\theta }=\min_{\theta }\sum_{s,d}{\left(\Delta A^{\left(s,d\right)}-k\cdot sens^{\left(s,d\right)}-b^{\left(d\right)}\right)}^{2}.$$ (5)

This is a quartic function with bound constraints, and thus can be solved by the typical nonlinear optimization algorithm such as gradient descent or branch and bound techniques [14]. Compared to the elastic net model, this model integrates different drugs into a unified framework, so similarities between different drugs, e.g., some BRAF or MEK inhibitors, could possibly be detected. Moreover, one sample treated with different drugs could be served as different samples according to our model, so the total number of samples is significantly increased, which helps alleviate the possibility of over fitting due to the limited existing data.

In this paper, the optimx package in R is used to implement the optimization. Optimx is an extension of the optim function, and quite suitable for the optimization of functions that are mostly smooth with parameters, and several or many of them are box constrained. The method "L-BFGS-B" is adopted, which is a limited-memory modification of the BFGS quasi-Newton method proposed by Byrd et al. [15]. The parameters $A_{j}^{m}$ and $A_{j}^{v}$ are constrained to [–5,5], $\rho_{j}^{\left(d\right)}\in [0,1],$ and *b* ^(d)^ ∈ [−20,20]. 10-fold cross validation is used to validate our method, where samples with respect to each drug are roughly divided into 10 equal parts. In each fold, one part is singled out as the test set, and the other nine parts serve as training data to get the best parameter set. After the iteration, Pearson correlation coefficient between predicted and observed sensitivities for each drug is used as the prediction performance of our model. We used the Wilcoxon rank-sum test to examine the relationships between drug sensitivities and drive gene mutations. In statistic theory, the Wilcoxon rank-sum test is a nonparametric two-sample test based solely on the order of observations from two samples.

### 2.3 Potential application to prediction of drug combination

Most human diseases are caused by complex biological processes and have more than one causing gene, so an individual drug is sometimes not enough to ensure a good clinical effect [16]. Experimentally, one can use a high-throughput screening technique to identify possible effective drug combinations [17], but the number of possible drug combinations increases exponentially with the increase of drugs. So a systematic computational scheme is expected. In drug combination model, we assume the inhibitor effects of different drugs are independent with each other. While the synergistic effect between two drugs is a common phenomenon. Considering that our model is based on the high-throughput data with several hundreds of cell lines and drugs, we believe that the independent assumption between different drugs will not seriously affected by the limited number of the possible synergistic effects.

In our model, effects of different drugs are integrated into a uniform framework giving it the possibility to predict the combination effect of different drugs. Suppose a sample is treated in turn by two drugs *d*_1_ and *d*_2_, and the effects by the different drugs are assumed to be independent. According to (2), the increased pathway activity after treatment by drug *d*_1_ is

$$AT(d_{1})=\sum_{i=1}^{12}\sum_{j}\left(m_{ij}A_{{}_{ij}}^{m}+v_{ij}A_{{}_{ij}}^{v}\right)\rho_{ij}^{\left(d_{1}\right)}\alpha^{\left(d_{1}\right)}.$$ (6)

After treatment of drug *d*_2_, the increased pathway activity is decreased to

$$AT(d_{1},d_{2})=\sum_{i=1}^{12}\sum_{j}\left(m_{ij}A_{{}_{ij}}^{m}+v_{ij}A_{{}_{ij}}^{v}\right)\rho_{ij}^{\left(d_{1}\right)}\alpha^{\left(d_{1}\right)}\rho_{ij}^{\left(d_{2}\right)}\alpha^{\left(d_{2}\right)}.$$ (7)

So the final activity reduction after combination treatment of *d*_1_ and *d*_2_ will be

$$\Delta A(d_{1},d_{2})=AI-AT(d_{1},d_{2})=\sum_{i=1}^{12}\sum_{j}\left(m_{ij}A_{{}_{ij}}^{m}+v_{ij}A_{{}_{ij}}^{v}\right)\left(1-\rho_{ij}^{\left(d_{1}\right)}\alpha^{\left(d_{1}\right)}\rho_{ij}^{\left(d_{2}\right)}\alpha^{\left(d_{2}\right)}\right).$$ (8)

For each sample, we can calculate the Δ*A*(*d*_1_,*d*_2_) for all possible drug combinations according to mutation and copy number statuses of cancer driver genes. Then top drug combinations can be served as potential therapies for treatment. Along this line, combination effects of more drugs can be modeled similarly.

## Result and Discussion

### 3.1 Mutation of cancer genes in MAPK pathway and their relations with drug sensitivity

**Fig 2:**
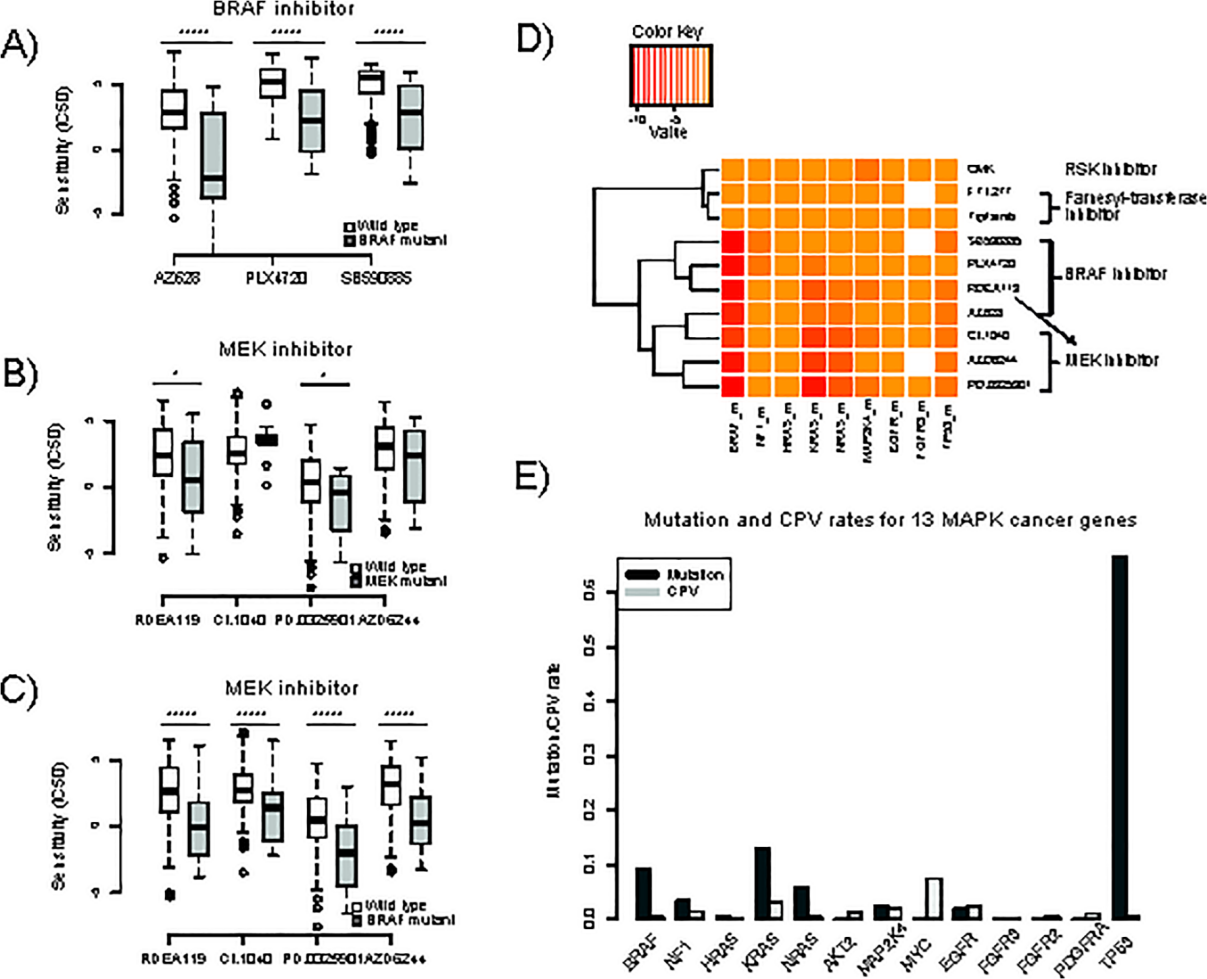
Mutation and drug sensitivity distributions of the CGP dataset. (A) Sensitivities of BRAF wild type and mutant samples to three BRAF inhibitors, i.e., AZ628, PLX4720 and SB590885. P-values by Wilcoxon Rank Sum test are 3.84e-6, 9.98e-10 and 1.33e-11, respectively. (B) Sensitivities of MEK wild type and mutant samples to four MEK inhibitors. (C) Sensitivities of BRAF wild type and mutant samples to four MEK inhibitors. (D) Drug sensitivity differences between wild type and mutant samples for cancer-related genes in MAPK pathway. Color key indicates log p-value between wild type and gene mutation samples by two-sample Wilcoxon Rank Sum test. (E) Mutation and CPV rates of 13 cancer-related genes in MAPK pathway.

We first examined the mutations of cancer genes in MAPK pathway and their relations with anticancer drug sensitivities. In Fig 2A, we showed sensitivity differences of three BRAF inhibitors, i.e., AZ628, PLX4720 and SB590885, at BRAF wild type and BRAF mutant samples. As is shown, all three BRAF inhibitors show significantly higher sensitivity against BRAF mutant samples, with p-values 3.84e-6, 9.98e-10 and 1.33e-11 by Wilcoxon Rank Sum test, respectively (Fig 2A). Next, we examined the above phenomenon for four MEK inhibitors. As is shown in Fig 2, MEK inhibitors, especially CI.1040, did not show much sensitivity difference between MEK mutant and wild type samples (p-values are 0.05, 0.16, 0.07 and 0.24, Fig 2B), but have significantly high sensitivity for BRAF mutant samples (p-values are 5.18e-10, 1.02e-5, 2.70e-10, and 5.01e-8, Fig 2C). This phenomenon reconfirms that BRAF mutation is a strong predictor of sensitivity to MEK inhibitors [9, 18–20]. From a network flow viewpoint, one possible explanation for this phenomenon is the specific topological structure of the MAPK signal pathway. In the MAPK pathway, BRAF lies upstream of MEK, and every signaling path from BRAF will flow to MEK. So if there were a flow increase due to BRAF mutation, the increased activity would finally flow to its downstream nodes. MEK inhibitors should have a good performance because MEK is served as the “hub” node in the sub-network induced by BRAF and its connected nodes. The above explanation reminds us a flow-based scheme to predict drug sensitivity by making a full use of pathway topological structure.

To have an overall picture of the relationships between drug sensitivities and driver gene mutations, in Fig 2D, we show the difference in drug sensitivities between wild type and mutant samples for all MAPK driver genes and drugs. The color of key indicates log p-value of wild type and gene mutation samples by two-sample Wilcoxon Rank Sum test. Only 9 cancer genes are displayed because the other4 genes, i.e., AKT2, MYC, FGFR2 and PDGFRA, have no mutant samples in the selected dataset (Fig 2E). Missing values indicate that there is no mutant sample for the corresponding drug treatment. As is shown in Fig 2D, ten MAPK target drugs form two distinct clusters. The first cluster consists of 3 drugs, one is an RSK inhibitor, and the other two are Farnesyl-transferase inhibitors. Drugs in this cluster show no significant difference in sensitivity between mutant and wild type samples for all cancer driver genes. The remaining 7 drugs, i.e., 3 BRAF inhibitors and 4 MEK inhibitors, form the second cluster. These 7 drugs also constitute two fairly separate subclusters, with an exception of REDA119, which is a MEK inhibitor but much closer to BRAF inhibitors in the heat map. As to each gene, we find that BRAF mutant and wild type samples show the most significant sensitivity difference for most drugs compared to other driver genes, which reconfirms the observation that mutation of BRAF is the strongest predictor of drug sensitivity for MEK and BRAF inhibitors. TP53 mutant cell lines are also prone to be more sensitivity to MEK and BRAF inhibitors, but the phenomenon is not so strong as BRAF according to the heat map. Mutations of KRAS and NRAS also have strong effect for some BRAF and MEK inhibitors, but influence patterns are quite different, e.g., samples with KRAS mutation are more sensitive to MEK inhibitor. There are also some genes, such as HRAS, EGFR and FGFR3, whose mutation samples are not sensitive to any drug, which means that mutations of these genes have less contribution to the pathway activity or the increased activities cannot be reduced by these 10 MAPK drugs.

### 3.2 Drug sensitivity prediction for the MAPK pathway

Due to the large sample and variable size, the optimization of Eq (5) for the entire CGP dataset is difficult to solve. To alleviate the problem, in this section, we restricted our analysis to cancer related genes and drugs targeting only in the MAPK pathway. The MAPKs are a group of protein serine/threonine kinases that are activated in response to a variety of extracellular stimuli and mediate signal transduction from the cell surface to the nucleus [21]. In combination with several other signaling pathways, they can differentially alter phosphorylation status of numerous proteins, including transcription factors, cytoskeletal proteins, kinases and other enzymes, and greatly influence gene expression, metabolism, cell division, cell morphology and cell survival [22–24]. Epigenetic aberrations of these enzymes or of the signaling cascades that regulate them have been implicated in a variety of human diseases including cancer, inflammation, and cardiovascular disease [25]. According to Table 1, the MAPK pathway consists of 256 genes and 875 interactions. Of the 63 cancer driver genes, 13 are included in this pathway. As is shown in Table 2, there are 10 anticancer drugs that target specifically to genes from the MAPK pathway. Among these 10 drugs, 3 of them (AZ628, PLX4720 and SB590885) target BRAF, 4 of them (RDEA119, CI-1040, PD-0325901 and AZD6244) target MEK, CMK targets RSK, and the remaining two (FTI-277 and Tipifarnib) inhibit Farnesyl-transferases.

**Table 2:** 10 drugs which target genes in MAPK pathway.

Drug ID	Drug name	Drug Target(s)	No. Cell lines screened
29	AZ628	BRAF	293
64	CMK	RSK	289
166	FTI-277	Farnesyl-transferase (FNTA)	319
204	Tipifarnib	Farnesyl-transferase (FNTA)	338
1014	RDEA119	MEK1/2	469
1015	CI-1040	MEK1/2	431
1036	PLX4720	BRAF	448
1060	PD-0325901	MEK1/2	469
1061	SB590885	BRAF	455
1062	AZD6244	MEK1/2	445

When looking at cancer genes and drugs in only MAPK pathway, mutation and copy number alteration of cancer genes from other pathways will eliminate from the formula since their activity contributions to these pathways are equal before and after drug treatment. So the Eq (5) can be simplified as

$$\hat{\theta }=\min_{\theta }\sum_{s,d=1}^{10}(\sum_{j=1}^{13}\left(m_{j}^{\left(s\right)}A_{j}^{m}+v_{j}^{\left(s\right)}A_{j}^{v}\right)\left(1-\rho_{j}^{\left(d\right)}\alpha^{\left(d\right)}\right){-sens^{\left(s,d\right)}-b^{\left(d\right)})}^{2}$$ (9)

In this formula, mutation and CPV status of each gene and sensitivities of 10 drugs to each sample are known, and all other variables are to be determined. Since there are 13 driver genes and 10 drugs, the total number of free variables is (13+13) + 13 * 10 + 10 = 166. According to our model, one cell line treated by different drugs is considered to be different. Taking all drugs and cell lines into consideration, the total number of samples increases to 3,957, which is much more than the variables. So the optimization is expected to be much reliable.

**Fig 3:**
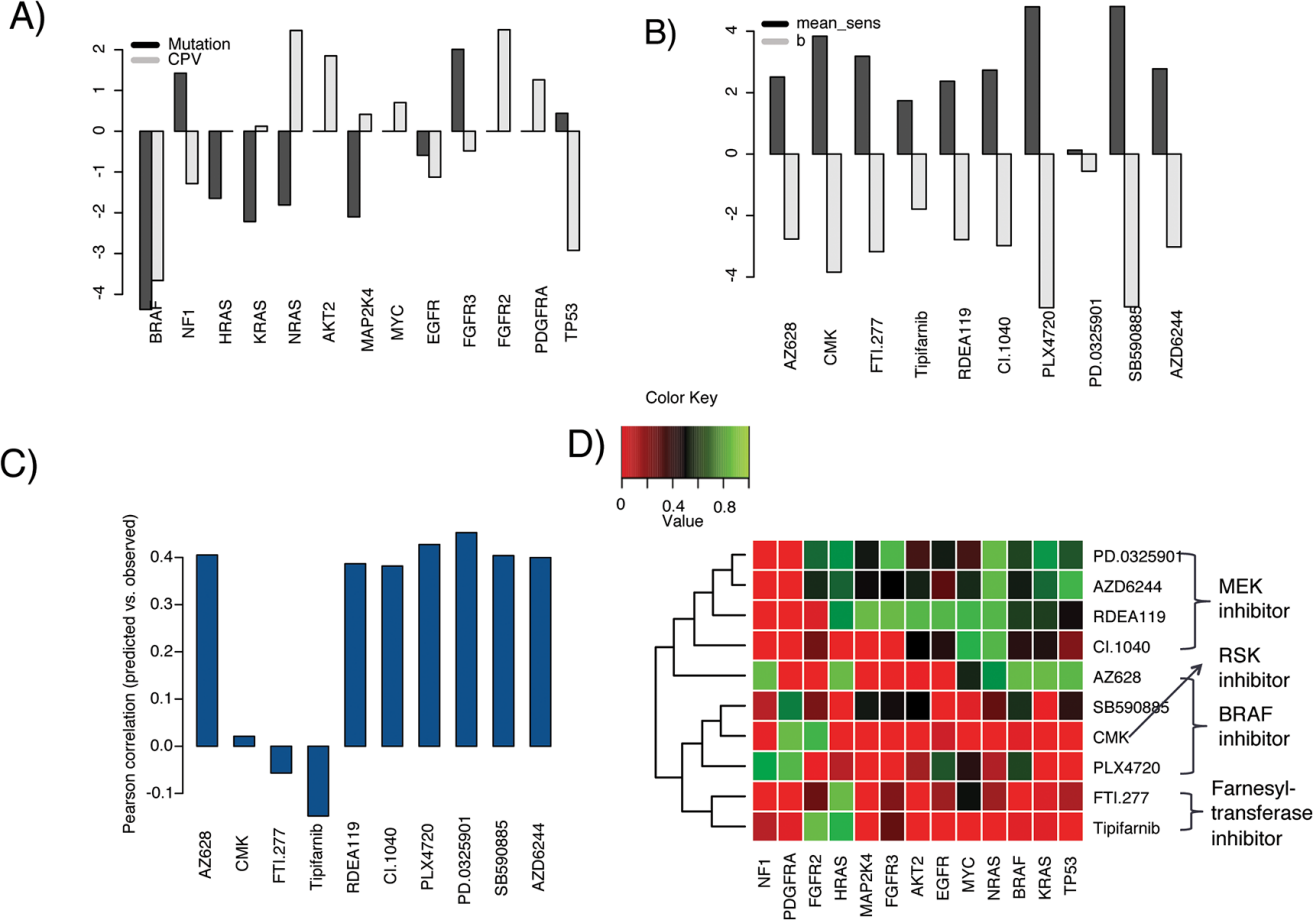
Prediction results of the network-flow model. A) Contributions of different genetic alterations (mutation and copy number variance) to the MAPK pathway activity; B) Predicted residue *b* is consistent with the mean drug sensitivities (IC50); C) Pearson correlation coefficients between predicted and real MAPK drug sensitivities; D) Predicted drug effects of 10 MAPK drugs to 13 cancer-related genes.

By running Optimx, the sum of squared errors is minimized to 12,471.64, with an averaged error 1.7753 for each sample. Increased pathway activities induced by mutations of different driver genes are shown in Fig 3A. As is shown, mutation and copy number alternation of BRAF have the largest negative coefficient in regression of drug response (IC50). Known that small IC50 values indicate high sensitivity of the drug, the above phenomenon showed that BRAF is the greatest contributor to MAPK pathway activity and drug sensitivity, which is consistent with the observation from Fig 2D. Other important contributors to MAPK pathway activity include KRAS, NRAS and MAP2K4, and all of them have positive contribution to drug sensitivity. Two other genes, NF1 and FGFR3, show negative contribution to drug sensitivity. This phenomenon for NF1 is reasonable knowing that NF1 is an inhibitor of RAS proteins including KRAS, NRAS, and HRAS et al. Meanwhile, mutation of FGFR3 also has a high negative contribution to drug sensitivity, but such contribution is not so reliable since there are only two FGFR3 mutant cell lines, in such case parameter estimation could be seriously affected by random noise.

Pearson correlation coefficients between predicted and observed sensitivities for different drugs are shown in Fig 3C. Interestingly, all 3 BRAF inhibitors and 4 MEK inhibitors achieve Pearson correlations around 0.4, which is comparable to the results by elastic net [9]. But in elastic net model, mutation, copy number variance, and a notably huge dimension of gene expression data are merged together as the input feature for each drug, so the total number of features is too high compared to the limited number of samples. Therefore, the final selected features may have the possibility of over fitting by including some false positive features just by chance. But our model is based only on a small fraction of gene mutation and copy number alteration features, so the result is quite promising even slightly lower than the elastic net regression. Besides BRAF and MEK inhibitors, our model gets very low accuracy for two farnesyl-transferase inhibitors (FTIs), FTI-277 and Tipifarnib. FTIs were initially developed to target RAS proteins because farnesyl is necessary to attach RAS to the cell membrane. Without attachment to the cell membrane, RAS is not able to transfer signals from membrane receptors [26]. But it has been reported recently that geranylgeranyl-transferase (GGTase) modification is an alternative route to creation of biologically active RAS other than farnesyl-transferase [26, 27]. Meanwhile, some other proteins other than RAS may be also modified by farnesyl-transferase, and thus can be inhibited by farnesyl-transferase inhibitors [26]. In a word, these two drugs are neither quite sensitive nor specific to RAS proteins, thus cannot be predicted well by our model.

Predicted drug effects of 10 MAPK drugs to 13 cancer driver genes are shown as Fig 3D. This heat map seems quite similar with Fig 2 in terms of the overall structure, i.e., BRAF, MEK and farnesyl-transferase inhibitors are clustered together. Interestingly, we found that influence effects of different driver genes to different BRAF or MEK inhibitors are significantly different (p-values are 0.022 between CI.1040 and PD.0325901, 0.028 between CI.1040 and AZD6244, by paired t-test). For example, four MEK inhibitors all have strong positive drug effect to NRAS mutation, but have no effect on NF1 and PDGFRA. Different MEK inhibitors also have specific drug effect genes. For example, PD.0325901 and RDEA119 have strong positive effects on FGFR3, but other two MAK inhibitors, i.e., AZD6244 and CI.1040 do not show high effects to it, especially CI.1040. This may be attributed to their different inhibition mechanisms. Although sharing the same target gene, they may target different active regions. Two farnesyl-transferase inhibitors show very similar drug effect patterns. They only have high positive drug affect on mutation of HRAS, one of their potential target genes. Additionally, there are also some drugs that could affect the downstream genes of their target genes, such as AZ628, a BRAF inhibitor, which has strong positive effect on TP53 and RAS proteins (KRAS, HRAS and NRAS). This may be due to the bias of the data, e.g., numbers of mutant samples treated by this drug are very limited, there could be some unknown interactions among these genes such as cofactors, or recruited by the same proteins.

### 3.3. Effects by different drug combinations

Since our model integrated all drug effects into a unified model, it can possibly predict combination effects of different drugs. The underlying assumption is that effects by different drugs are independent with each other, so activity contributions by different drugs can be calculated separately and then merged together. For each sample in the CGP dataset, we computed combination effects of all possible pairs of MAPK targeting drugs (results are shown in S1 File), and a case study for 647-V cell line is shown in Table 3. This cell line has 2 somatic mutations (MAP2K4 and TP53), and a gene copy number alteration (MAP2K4). The top-20 and bottom-20 drug combinations by effectiveness are shown in Table 3. Results from the two farnesyl-transferase inhibitors and CMK are discarded due to their poor cross-validation accuracy, so the top potentially effective drug combinations are RDEA119 and PLX4720, the inhibitors of MEK and BRAF, respectively. But combination of two BRAF inhibitors, i.e., AZ628 and PLX4720, has a very poor final effect. This shows that a combination of drugs targeting different genes could be potentially more effective than single-target drugs. However, we cannot declare that every possible combination of MEK and BRAF inhibitors could work. An example is AZD6244 and PLX4720, which is also a combination of MEK and BRAF inhibitors, but shows very poor combination effect

**Table 3:** Drug combination effects for 647-V cell line.

Top-20 drug combinations			Bottom-20 drug combinations		
Drug 1	Drug 2	Comb_Effect	Drug 1	Drug 2	Comb_Effect
CMK	RDEA119	-1.474633024	CMK	AZD6244	-0.436698712
Tipifarnib	RDEA119	-1.474633024	Tipifarnib	AZD6244	-0.436698712
RDEA119	PLX4720	-1.474633024	PLX4720	AZD6244	-0.436698712
FTI.277	RDEA119	-1.43616255	FTI.277	AZD6244	-0.428415918
RDEA119	CI.1040	-1.41783194	CI.1040	AZD6244	-0.42446929
RDEA119	SB590885	-1.381601018	AZ628	AZD6244	-0.389438855
RDEA119	PD.0325901	-1.323177439	CMK	Tipifarnib	0
RDEA119	AZD6244	-1.271828928	CMK	PLX4720	0
AZ628	RDEA119	-1.255128688	Tipifarnib	PLX4720	0
PD.0325901	SB590885	-0.965212144	CMK	FTI.277	0.073743397
SB590885	AZD6244	-0.874509433	FTI.277	Tipifarnib	0.073743397
PD.0325901	AZD6244	-0.861293939	FTI.277	PLX4720	0.073743397
CMK	SB590885	-0.718299199	CMK	CI.1040	0.108881031
Tipifarnib	SB590885	-0.718299199	Tipifarnib	CI.1040	0.108881031
PLX4720	SB590885	-0.718299199	CI.1040	PLX4720	0.108881031
FTI.277	SB590885	-0.674811232	FTI.277	CI.1040	0.16439154
CI.1040	SB590885	-0.654089861	AZ628	CMK	0.420764127
CMK	PD.0325901	-0.605061038	AZ628	Tipifarnib	0.420764127
Tipifarnib	PD.0325901	-0.605061038	AZ628	PLX4720	0.420764127
PLX4720	PD.0325901	-0.605061038	AZ628	FTI.277	0.424047635

## Conclusion

Pathway activity system is in a stable state for normal cells, but if a cancer driver gene is mutant or has copy number alteration in chromosome, the whole system will be disturbed. In this paper, a network-flow based model is proposed to capture this phenomenon. In our model, mutation and copy number variance of a cancer related gene are assumed to have a perturbation to the system, and anticancer drugs could affect the stability of the system by reducing its pathway activity flowing through their target genes. Based on above hypotheses, we come up with a systematic framework to predict drug sensitivity based on network-flow theory and a nonlinear optimization algorithm. Through training our model on drugs and cancer driver genes of the MAPK signaling pathway, we obtained sensitivity contributions of different driver genes to different anti-cancer drugs, and different drug effects to different cancer related genes. The obtained results are consistent with previous analyses and literature reports. We found that although many drugs inhibiting the same target genes, they may demonstrate apparently different drug effects. By a 10-fold cross-validation, we obtained Pearson correlation coefficients of predicted and observed IC50 around 0.4 for all BRAF and MEK inhibitors, which is comparable with the elastic net regression. However, instead of using a huge number of genomic features including transcription profiling, mutation, and copy number statuses, our model is only based on the mutation and copy number statuses of cancer driver genes in MAPK pathway. So our result is fairly promising and has better generalization ability. In addition, since effects of different drugs are integrated into a unified model, our model could predict drug combination effects based on the optimized parameters.

However, our model has some limitations. First, due to the limited number of samples, prediction accuracies for some drugs are still not very satisfactory. Second, different mutation types of one cancer gene may have different contributions to the pathway activity, but they are treated equally in our model. For example, it is reported that different KRAS mutations may lead to a different signal transduction cascade in NSCLC and to a different carcinogenesis and drug sensitivity to EGFR inhibitors [27]. Third, besides mutation and copy number alterations, gene expression profiles may also have predictive power in determination of drug effects, and thus need to be incorporated to our model. Fourth, our research mainly focuses on exonic mutations provided by CGP. However, intronic and intergenetic mutations may also play a role in aberrations of gene regulation. So some important biomarkers will probably be lost without these kinds of data. Fifth, when predicting the affects by different drug combinations we assume that the effects by different drugs are independent with each other, which is not the case in cancer treatments. So a better prediction result would be achieved if synergistic effects of drugs were incorporated into the model.

## Supporting Information

S1 File Predicted drug combination effects of all cell lines in CGP. (ZIP)

S1 Table Mutation, copy number and drug response data in CGP dataset. (XLSX)
